# Functional Adaptation in Female Rats: The Role of Estrogen Signaling

**DOI:** 10.1371/journal.pone.0043215

**Published:** 2012-09-11

**Authors:** Susannah J. Sample, Molly A. Racette, Zhengling Hao, Cathy F. Thomas, Mary Behan, Peter Muir

**Affiliations:** 1 Comparative Orthopaedic Research Laboratory, School of Veterinary Medicine, University of Wisconsin-Madison, Madison, Wisconsin, United States of America; 2 Department of Comparative Biosciences, School of Veterinary Medicine, University of Wisconsin-Madison, Madison, Wisconsin, United States of America; Oklahoma State University, United States of America

## Abstract

**Background:**

Sex steroids have direct effects on the skeleton. Estrogen acts on the skeleton via the classical genomic estrogen receptors alpha and beta (ERα and ERβ), a membrane ER, and the non-genomic G-protein coupled estrogen receptor (GPER). GPER is distributed throughout the nervous system, but little is known about its effects on bone. In male rats, adaptation to loading is neuronally regulated, but this has not been studied in females.

**Methodology/Principal Findings:**

We used the rat ulna end-loading model to induce an adaptive modeling response in ovariectomized (OVX) female Sprague-Dawley rats. Rats were treated with a placebo, estrogen (17β-estradiol), or G-1, a GPER-specific agonist. Fourteen days after OVX, rats underwent unilateral cyclic loading of the right ulna; half of the rats in each group had brachial plexus anesthesia (BPA) of the loaded limb before loading. Ten days after loading, serum estrogen concentrations, dorsal root ganglion (DRG) gene expression of ERα, ERβ, GPER, CGRPα, TRPV1, TRPV4 and TRPA1, and load-induced skeletal responses were quantified. We hypothesized that estrogen and G-1 treatment would influence skeletal responses to cyclic loading through a neuronal mechanism. We found that estrogen suppresses periosteal bone formation in female rats. This physiological effect is not GPER-mediated. We also found that absolute mechanosensitivity in female rats was decreased, when compared with male rats. Blocking of adaptive bone formation by BPA in Placebo OVX females was reduced.

**Conclusions:**

Estrogen acts to decrease periosteal bone formation in female rats *in vivo*. This effect is not GPER-mediated. Gender differences in absolute bone mechanosensitivity exist in young Sprague-Dawley rats with reduced mechanosensitivity in females, although underlying bone formation rate associated with growth likely influences this observation. In contrast to female and male rats, central neuronal signals had a diminished effect on adaptive bone formation in estrogen-deficient female rats.

## Introduction

With 2 million fractures and associated health care costs of $17 billion currently, the economic cost of osteoporosis is expected to rise 50% by 2025 [1]. Understanding how estrogen and estrogen receptors contribute to the failure of functional adaptation in osteoporosis would enhance the management of this condition [2]. The skeleton is exquisitely sensitive to loading and functional adaptation occurs in response to minimal cyclic load and strain [3]. However, mechanosensing signaling pathways in bone are not clearly defined.

It is widely accepted that the osteocyte is the primary mechanosensory cell in bone. Detection of mechanical strain by osteocytes fits well with the view that skeletal adaptation is a local phenomenon. In the past, nerve endings in bone have not been considered a functionally important regulator of mechanotransduction. However, recent data suggests that the nervous system is involved in the regulation of skeletal adaptation [4]–[6]. Unmyelinated sensory nerves establish direct connections between individual bone cells and the brain [7], potentially enabling direct neural regulation of bone physiology. The periosteum is innervated with a dense meshwork of nerve fibers optimized for detection of mechanical distortion [8], and contains sensory nerves that release a range of neuropeptides and neurotransmitters, including calcitonin gene-related peptide (CGRP) and glutamate [9], [10]. Sensory fibers that innervate bone contain a phenotypically restricted set of neurotransmitters in which the peptidergic neurotransmitters substance P and CGRP are enriched [11]. Site-specific sprouting of CGRP fibers coincides with bone formation and modeling during fracture healing [12]. Afferent sensory nerve fibers are also a potential means by which the nervous system may detect loading events within the skeleton. Voltage-gated channels, such as transient receptor potential vanilloid-1 and -4 (TRPV1 and TRPV4) and transient receptor potential ankyrin-1 (TRPA1) channels, are abundent in primary afferent nocipetive neurons and detect peripheral stimuli, such as changes in tissue pH and mechanical distortion [13]–[15]. TRPV4^−/−^ and TRPA1^−/−^ mice exhibit reduced action potential firing in response to mechanical stimulation of skin [13], [15]. Such findings suggest a potential role for these receptors in bone mechanosensing.

It is well established that sex steroids have important effects on the skeleton. Osteoporosis is characterized by an increase in bone resorption relative to bone formation, resulting in low bone mass and a reduced resistance to fracture. In addition, loss of estrogen induces a dramatic and specific reduction in the density of nerve fibers in bones that lose bone mass after ovariectomy (OVX) [16]. Estrogen acts on the skeleton via the classical genomic estrogen receptors –alpha and –beta (ERα and ERβ). Of these receptors, ERα is believed to be the primary mediator of estrogen's action in bone [17]. ERα knockout mice have a decreased adaptive response to bone loading [18]. In female ERβ knockout mice, the opposite effect is found [19].

Estrogen also has rapid signaling effects by acting on a membrane ER and a G-protein-coupled estrogen receptor (GPER, also known as GPR30) [20]–[22]. GPER is widely distributed in the brain, spinal cord, and dorsal root ganglion (DRG) sensory neurons [23]–[25]. Classical ER antagonists, such as tamoxifen or ICI 182780, are GPER agonists [22], [26]. The action of estrogen on GPER in sensory neurons induces mechanical hyperalgesia [25]. Therefore, GPER signaling may modify mechanosensing of peripheral stimuli. In the present study, we used the ulna end-loading model in ovariectomized (OVX) female rats to study the effects of estrogen and GPER signaling on the neuronal regulation of bone adaptation to mechanical loading. Our goal was to determine whether the neuronal regulation of adaptation to mechanical loading is estrogen-dependent in female rats.

## Materials and Methods

### Animals

A homogeneous group of 48 female Sprague-Dawley rats (body weight 244–284 g, aged 118±14 days) was used for the study. Rats were provided with food and water ad libitum. OVX and ulna loading was performed under isoflurane anesthesia with butorphanol analgesia. Humane euthanasia was performed under isoflurane anesthesia at the end of the experimental period.

### Ethics statement

All procedures were performed in strict accordance with the recommendations in the Guide for the Care and Use of Laboratory Animals of the National Institutes of Health and the American Veterinary Medical Association and with approval from the Animal Care Committee of the University of Wisconsin-Madison (V1148).

### Experimental design

Rats underwent OVX and were randomly assigned to 3 groups (16 rats/group), based on the contents of a subcutaneous pellet implanted immediately after OVX: Placebo, Estrogen (17β-estradiol), or G-1, a GPER-specific agonist [26]. Fourteen days after OVX and pellet implantation, the right ulna of each rat was cyclically loaded; half the rats from each group received brachial plexus anesthesia (BPA) of the loaded limb before loading (Placebo+BPA, Estrogen+BPA, G-1+BPA), while the remaining rats were loaded without BPA (Placebo, Estrogen, G-1). All rats received an intra-peritoneal injection of calcein green (7 mg/kg) at the time of loading, and a subcutaneous injection of alizarin red (30 mg/kg) 7 days later. Rats were euthanatized 10 days after loading.

### Ovariectomy and pellet implantation

OVX and pellet implantation were performed under isoflurane-induced general anesthesia. Animals underwent bilateral flank OVX. After completion of OVX, a pellet containing either G-1 (0.32 mg/day) [27], Estradiol 17β (4.1 µg/day) [28], or placebo (0.32 mg/day) (Innovative Research of America, Sarasota, FL) was implanted subcutaneously dorsally between the scapulae.

### In-vivo ulnar loading

**Figure 1:**
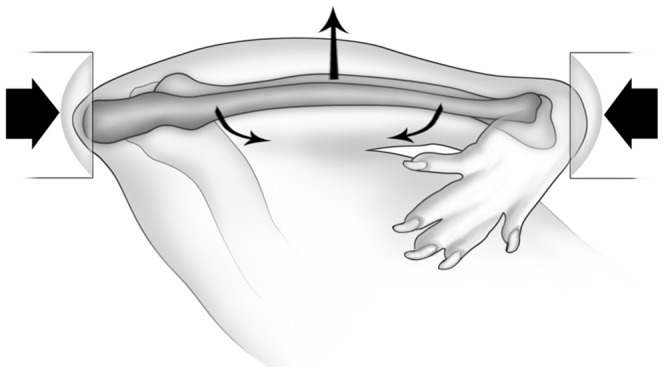
Schematic diagram of the rat ulna loading model. The antebrachium was placed horizontally in loading cups attached to a materials testing machine. The medio-lateral diaphyseal curvature of the rat ulna is accentuated through axial compression, most of which is translated into a bending moment, which is greatest at ∼60% of the total bone length measured from the proximal end of the ulna [30]. Reproduced from [5] with permission from John Wiley & Sons.

*In-vivo* loading of the right ulna was performed under isoflurane-induced general anesthesia. The right antebrachium of each rat was placed horizontally between two loading cups, which were fixed to the loading platen and actuator of a materials testing machine (Model 8800 DynaMight; Instron, Canton, MA, USA) with a 250N load cell (Honeywell Sensotec, Canton, MA, USA). The right ulna then underwent cyclic loading by means of axial compression, which accentuates the pre-existing mediolateral curvature of the diaphysis of the rat ulna, translating most of the axial force into a bending moment (**Fig. 1**). To determine the relationship between peak load and initial peak strain for female rats using this model, we performed an ex-vivo study using four rats. A single rosette strain gage (EA-06-031DE-120, 120Ω; Vishay Micromeasurements, Malvern, PA, USA) was bonded to the diaphysis of the caudal medial surface of the right ulna at 60% of bone length from the proximal end of the bone. The right ulna was cyclically end-loaded in compression at 4 Hz for a small number of cycles (50 cycles) using a series of compressive loads [4]. As a result of these strain gage data, the rats in this study were loaded at −17N for 1,500 cycles using a 2 Hz haversine wave. This peak load resulted in peak compressive strains of approximately −3,500 µe at 60% total bone length measured from the proximal end of the ulna.

### Brachial Plexus Anesthesia

Perineural anesthesia of the nerves of the right brachial plexus was performed 5 min before loading using bupivicaine (Marcaine 0.5%; Hospira, Lake Forest, IL, USA) at a dose of 2 mg/kg. A train-of-four nerve stimulator (Micro Stim; Neuro Technology, Houston, TX, USA) was used to confirm correct positioning of the insulated injection needle (ProBloc II; Portex, Smiths Medical, St Paul, MN, USA). Functional blocking of neuronal signaling between the spinal cord and the loaded limb was confirmed by observing temporary paralysis of the limb on recovery from anesthesia, which resolved within 2 hours of loading.

### Quantification of dorsal root ganglion ERα, ERβ, GPER, CGRPα, TRPV1, TRPV4, and TRPA1 gene expression

During euthanasia, rats were anesthetized with isoflurane. A heparinized blood sample was collected, and heparin was then injected into the left ventricle (0.1 ml, 1,000 iu/ml). Rats were perfused with 200 ml of saline followed by RNAlater solution (Ambion, Foster City, CA). Left and right DRG from the brachial intumescence (C_6_-T_2_) and trigeminal ganglia were dissected and stored in RNAlater solution at −80°C for analysis. ERα, ERβ, GPER, CGRPα, TRPV1, TRPV4, and TRPA1 expression were determined by qRT-PCR. Total RNA was further purified using a RNA clean-up kit (Qiagen, Valencia, CA). cDNA was generated from 0.2 to 2 mg of total RNA by using the superscript III first-strand synthesis system for reverse-transcriptase-polymerase chain reaction (RT-PCR) (Invitrogen, Carlsbad, CA). qRT-PCR was performed using standard methods and SYBR green methodology using a Bio-Rad thermocycler (MyiQ and IQ-SYBR Green Supermix, Bio-Rad, Hercules, CA) according to the manufacturer's instructions. Oligonucleotide primers purchased for the following genes (Integrated DNA Technologies, Coralville, IA): ERα, ERβ, GPER, CGRPα, TRPV1, TRPV4, and TRPA1 were used for PCR (**Table 1**). For ERα, ERβ, and GPER gene expression, the 18S rRNA gene was used as the housekeeping gene; for CGRPα, TRPV1, TRPV4, and TRPA1 gene expression, the protein gene product 9.5 (PGP9.5) gene was used as the housekeeping gene [29]. All PCR reactions were carried out in a final volume of 25 µl, which contained 12.5 µl of 2×SYBR Green (Bio- Rad, Hercules, CA), 1 µl of 5 µM forward primer, 1 µl of 5 µM reverse primer, 1 µl of cDNA and 9.5 µl of DEPC water. PCR cycling conditions were 2 min at 50°C, 8.5 min at 95°C, and 40 cycles of 95°C for 15 s, 60°C for 1 min and 1 min at 95°C, 1 min at 55°C. Assays were validated by the use of a no template control.

**Table 1:** Oligonucleotide primers for quantitative real-time reverse-transcriptase-polymerase chain reaction.

mRNA Targets	Primer Type	Olignonucleotides (5′ to 3′)	Amplicon Size (bp)	Sequence Reference
ERα	Forward	CAAACCAATGCACCATCGATAA	101	Hou et al. 2010 [47]
	Reverse	TTTTCGTATCCCGCCTTTCA		
ERβ	Forward	CTGTGTGGCCATAAAATCAACCT	101	Hou et al. 2010 [47]
	Reverse	AGGCAGGAATGCGAAATGAG		
GPER	Forward	TCTTCATCAGCGTCCACCTAC	172	Kuhn et al. 2008 [25]
	Reverse	TTGTCCCTGAAGGTCTCTCC		
CGRPα	Forward	GCATGGCCACTCTCAGTGAAG	77	Laboratory of Dr. Muir
	Reverse	CCTGACTTTCATCTGCATATAGTTCTG		U. Wisconsin-Madison
TRPV1	Forward	GGTGTGCCTGCACCTAGC	82	Zhao et al. 2010 [29]
	Reverse	CTCTTGGGGTGGGGACTC		
TRPV4	Forward	AGAAAGCGCCCATGGATT	102	Yang et al. 2006 [48]
	Reverse	TGGCTGCTTCTCTACGACCT		
PGP9.5	Forward	CCTGCTGCTGCTGTTTCC	107	Zhao et al. 2010 [29]
	Reverse	TGTCCCTTCAGTTCCTCAATTT		
18S rRNA	Forward	CGCCGCTAGAGGTGAAATTCT	100	Laboratory of Dr. Svaren
	Reverse	CGAACCTCCGACTTTCGTTCT		U. Wisconsin-Madison

**Note**: ERα and -β – estrogen receptors alpha and beta; GPER – G-protein coupled estrogen receptor; CGRPα – calcitonin gene-related peptide alpha; TRPV1 and −4 – transient receptor potential vanilloid-1 and −4; TRPA1 – transient receptor potential ankyrin-1; PGP9.5 – protein gene product 9.5.

### Quantification of plasma estrogen

Plasma estrogen levels were quantified by ELISA (IBL-America, Minneapolis).

### Bone histomorphometry

After euthanasia, pairs of ulnae and humeri were dissected along with surrounding tissue. Bones were dehydrated in ethanol (70% and then 100%), and embedded in methylmethacrylate. Transverse calcified sections, 125 µm thick, were made and mounted on standard microscope slides. Ulnae were sectioned at 60% of total bone length measured from the proximal end, where it has been shown maximal adaptation occurs with this model [30]. Humeri were sectioned at the mid-diaphysis. Confocal microscopy (MRC-1024 Laser Scanning Confocal Microscope; Bio-Rad, Hercules, CA, USA) was used to collect fluorescent images of each bone section. Both classical morphometric analysis and direct quantification of labeled new bone formation were used (Image J; NIH). Morphometric analysis included periosteal and endosteal mineralizing surface (MS/BS, %), mineral apposition rate (MAR, µm/day), and bone formation rate (BFR/BS, µm^3^/µm^2^/yr). Relative (R-L) rMS/BS, rMAR, and rBFR/BS were also calculated for the periosteal and endosteal surfaces. To compare the relative osteogenic response in each group with previous data in young male Sprague-Dawley rats, periosteal labeled bone formation was also directly measured and periosteal labeled bone area (Ps.L.B.Ar, %), as a percent of original cortical area, was determined, together with relative (R-L) rPs.L.B.Ar. for the Estrogen, Placebo and G-1 groups. Data from the present study were compared with previous work from our laboratory using male rats that underwent a similar loading protocol using the ulna end-loading model [4]. These male rats were loaded for 1,500 cycles with −18N at 4 Hz resulting in −3,750 µε at 60% total bone length measured from the proximal ulna [4]. Viscoelasticity effects in the ulna loading model [31] suggest that the applied cyclic load is similar between males and females. All measurements were made by a single observer (MR).

### Statistical analysis

For analysis of gene expression data, the threshold cycle values (Ct values) obtained from the exponential region of the PCR amplification plot from triplicate trials were averaged together. Relative expression of the genes of interest was then calculated using the −ΔΔCt method and a standard curve to determine primer efficiency [32]. Gene expression in the DRG was normalized to an internal control tissue, the ipsilateral trigeminal ganglion, which does not provide appendicular sensory innervation.

Data are reported as mean ± standard deviation. The Kolmogorov-Smirnov test was used to confirm that data were normally distributed. Right and left limbs and DRG were treated as separate experiments. Within the Load groups and the BPA+Load groups, differences in bone formation and gene expression between treatments were examined using a one-way ANOVA with a Dunnett post-hoc test; the Estrogen group served as the control. The Student's *t* test for unpaired data was used to examine differences between Load and BPA+Load groups within a given treatment. A single-sample Student's *t* test was used to determine whether rMS/BS, rMAR, rBFR/BS, and rPs.L.B.Ar were significantly different from zero. Effect size (ES: the standard mean difference between Load and BPA+Load groups) was calculated using the Cohen's d method; effects sizes greater than 0.5 and 0.8 were considered moderate and large, respectively [33]. For comparison of bone formation in male and female rats, a one-way ANOVA with a Dunnett's post-hoc test was also used to examine differences in rPs.L.B.Ar (%/1000 µε) in the loaded ulna between groups. The blocking effect of BPA was also determined [4]. Data were also normalized to the underlying growth rate for analysis [34]. ANOVA and a post-hoc Tukey's test was used to compare age between groups. Results were considered significant at *p*<0.05.

## Results

No evidence of fatigue damage, including woven bone formation or the presence of microdamage, was found in any bone sections upon microscopic evaluation. A total of 7 rats were excluded from histopathologic analysis of bone formation for the following reasons: lack of fluorochrome uptake (5), premature euthanasia due to a self-induced skin wound (1), and incomplete BPA noted on recovery from anesthesia, as determined by the animals having motor function of the right thoracic limb upon recovery from general anesthesia (1). The latter two rats were not used for gene expression analysis.

### Estrogen replacement suppressed periosteal labeled bone formation

**Figure 2:**
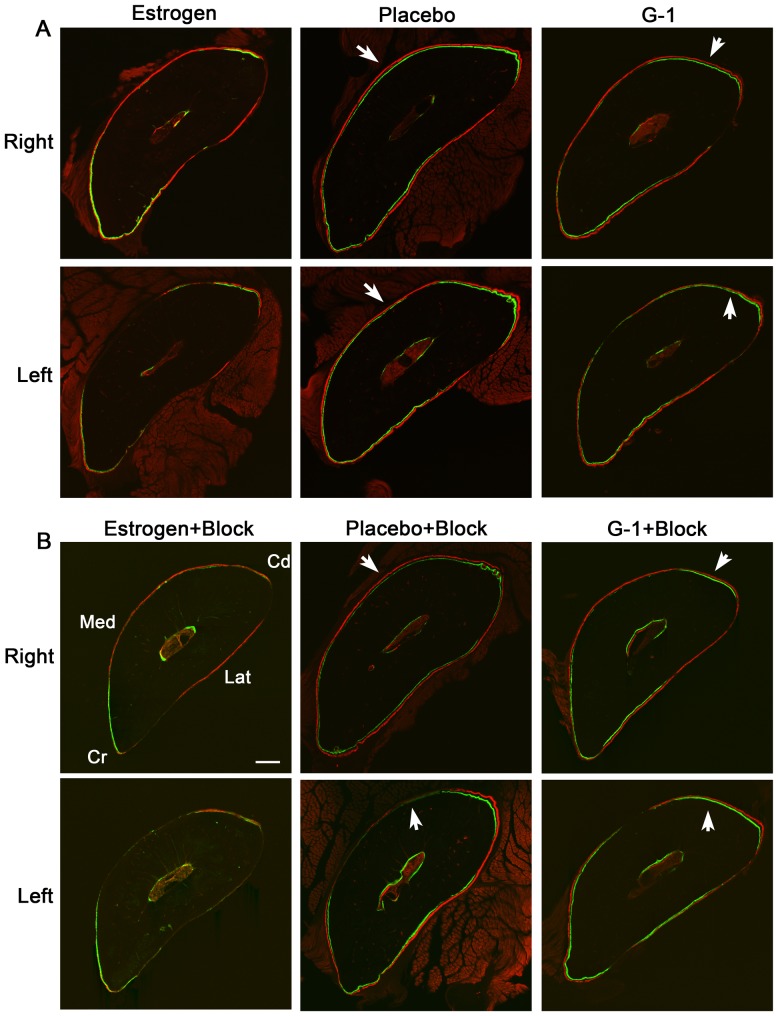
Labeled ulna bone formation was suppressed with estrogen treatment. Treatment with estrogen after ovariectomy (OVX) decreased load-induced labeled bone formation. (**A**) Rats treated with either Placebo or G-1, a GPER-specific agonist, had increased periosteal bone formation after right ulna loading, when compared to rats treated with estrogen. (**B**) Estrogen treatment in rats that underwent brachial plexus anesthesia (BPA) before loading of the right ulna also resulted in a decreased amount of periosteal bone formation, compared to placebo and G-1 treated rats. White arrows indicate periosteal labeled new bone formation. Bar = 250 µm. Cr, cranial; Cd, caudal; Med, medial; Lat, lateral. Estrogen group, n = 7; Placebo group n = 7; G-1 group, n = 6, Estrogen+BPA group, n = 8; Placebo+BPA group, n = 7; G-1+BPA group, n = 6.

Rats in the estrogen-deficient groups (Placebo and G-1) had significantly increased bone formation compared to rats in the Estrogen group (**Fig. 2**, **Table 2**). The Placebo group had increased Ps.MS/BS and Ps.BFR/BS in the loaded (*p*<0.05) and contralateral (*p*<0.001) ulnae, and both humeri (*p*<0.001) when compared to the Estrogen group; Ps.MAR in the Placebo group was also increased in the contralateral ulna (*p*<0.001) and both humeri (*p*<0.05) when compared to the Estrogen group. The G-1 group had increases in Ps.MS/BS and Ps.BFR/BS in the loaded (*p*<0.01) and contralateral (*p*<0.001) ulnae and both humeri (*p*<0.001) when compared to the Estrogen group; Ps.MAR of the G-1 group was also increased in the contralateral ulna (*p*<0.01) and the ipsilateral humerus (*p*<0.01) when compared to the Estrogen group. There were no significant differences in endosteal bone formation between the Placebo, Estrogen and G-1 groups.

**Table 2:** Periosteal bone formation after mechanical loading of the right ulna in ovariectomized female rats.

	Load Groups			BPA+Load Groups			Effect Size for BPA		
	*Estrogen*	*Placebo*	*G-1*	*Estrogen+BPA*	*Placebo+BPA*	*G-1+BPA*	*Estrogen*	*Placebo*	*G-1*
	(n = 7)	(n = 7)	(n = 6)	(n = 8)	(n = 7)	(n = 6)			
***Ulna***									
**Ps.MS/BS (%)**									
Right (loaded)	54.0±12.9	78.7±20.7*	86.8±8.9**	49.4±10.8	84.5±8.4 ***	86.8±7.4***	−0.38	0.37	0.00
Left	40.0±26.0	80.2±8.5***	81.7±7.2***	33.2±19.4	78.1±14.3***	66.0±19.9**	−0.30	−0.18	−1.04
rPs.MS/BS	13.9±25.2	−1.50±19.6	5.2±11.1	16.2±19.1#	6.4±14.0	20.8±21.9	0.10	0.46	0.90
**Ps.MAR (µm/day)**									
Right (loaded)	2.0±0.6	2.8±1.3	3.31±0.2	1.7±0.3	3.3±0.4***	3.1±0.4***	−0.66	0.50	−0.68
Left	1.5±0.9	3.1±0.4***	2.9±0.3**	1.3±0.7	3.1±0.5***	2.6±0.8**	−0.14	−0.11	−0.46
rPs.MAR	0.6±0.8	0.3±0.3	0.5±0.5*	0.40±0.7	0.2±0.5	0.5±0.8	−0.27	−0.24	0.00
**Ps.BFR/BS (µm^3^/µm^2^/yr)**									
Right (loaded)	421±230	887±424*	1048±138**	308±91	1028±205***	985±137***	−0.65	0.42	−0.46
Left	286±313	917±163***	848±72***	188±164	888±260***	669±346**	−0.39	−0.14	−0.71
rPs.BFR/BS	136±213	−30±446	200±113*	120±129#	140±246	316±362	−0.09	0.47	0.43
***Humerus***									
**Ps.MS/BS (%)**									
Right	13.2±6.5	46.9±5.9***	45.0±8.5***	16.0±16.9	36.5±7.9* a	41.3±16.6**	0.22	−1.50	−0.28
Left	17.9±15.8	43.2±6.6***	51.5±6.3***	15.0±11.0	40.9±8.5**	37.5±20.3*	−0.21	−0.30	−0.93
**Ps.MAR (µm/day)**									
Right	1.8±0.9	3.5±1.6*	3.8±0.4**	1.5±1.2	4.1±0.6***	3.4±0.9**	−0.27	0.46	−0.59
Left	1.8±2.5	4.3±0.9*	3.4±0.3	1.5±1.7	4.2±0.7**	3.1±1.9	−0.15	−0.14	−0.24
**Ps.BFR/BS (µm^3^/µm^2^/yr)**									
Right	97±75	623±284***	625±150***	142±218	544±173**	552±290**	0.28	−0.50	−0.32
Left	76±97	673±162***	646±94***	125±205	611±116**	532±405*	0.31	−0.44	−0.39

**Note**: Data represent mean ± standard deviation.

* *p*<0.05;

** *p*<0.01;

*** *p*<0.001 versus the estrogen treated group.

a *p*<0.05 versus the associated Load group that did not receive brachial plexus anesthesia (BPA) before loading.

# Significantly different from a hypothesized mean of zero (*p*<0.05). Effect size comparing Load and BPA+Load groups for each respective treatment. Ps.MS/BS - periosteal mineralizing surface; Ps.MAR - periosteal mineral apposition rate; Ps.BFR/BS - periosteal bone formation rate. Relative (R-L) values were also calculated – rPs.MS/BS, rPs.MAR, and rPs.BFR/BS.

Similar results were seen in the BPA groups (**Fig. 2** **,** **Table 2**). The Placebo+BPA group had increased Ps.MS/BS and Ps.BFR/BS in the loaded (*p*<0.001) and contralateral (*p*<0.001) ulnae, the ipsilateral humerus (*p*<0.05 for Ps.MS/BS; *p*<0.01 for Ps.BFR/BS), and the contralateral humerus (*p*<0.01), when compared to the Estrogen+BPA group; Ps.MAR of the Placebo+BPA group was also increased in the loaded and contralateral ulnae (*p*<0.001), ipsilateral humerus (*p*<0.001) and contralateral humerus (*p*<0.01), when compared to the Estrogen+BPA group. The G-1+BPA group had increased Ps.MS/BS and Ps.BFR/BS in the loaded ulna (*p*<0.001), contralateral ulna and ipsilateral humerus (*p*<0.01), and the contralateral humerus (Ps.BFR/BS *p*<0.05), when compared to the Estrogen+BPA group; Ps.MAR of the G-1+BPA group was increased in the loaded (*p*<0.001) and contralateral ulna (*p*<0.01) and the ipsilateral humerus (*p*<0.01), when compared to the Estrogen+BPA group. Significant increases in endosteal bone formation were also seen in the absence of estrogen (**Fig. 2** **,** **Table 3**). Compared to the Estrogen+BPA group, the following differences were noted: the Placebo+BPA group had increased En.MS/BS in the loaded ulna and both humeri (*p*<0.01), increased En.MAR and En.BFR/MS in the contralateral ulna (*p*<0.05), and increased En.MAR and En.BFR/BS in both humeri (*p*<0.001); the G-1+BPA group had increased En.MS/BS in the loaded ulna (*p*<0.05), En.MAR in the ipsilateral (*p*<0.05) and contralateral (*p*<0.01) humeri, and increased En.BFR/BS in both humeri (*p*<0.01).

**Table 3:** Endosteal bone formation after mechanical loading of the right ulna in ovariectomized female rats.

	Load Groups			BPA+Load Groups			Effect Size for BPA		
	*Estrogen*	*Placebo*	*G-1*	*Estrogen+BPA*	*Placebo+BPA*	*G-1+BPA*	*Estrogen*	*Placebo*	*G-1*
	(n = 7)	(n = 7)	(n = 6)	(n = 8)	(n = 7)	(n = 6)			
	***Ulna***								
**En.MS/BS (%)**									
Right (loaded)	17.7±17.1	33.3±24.9	23.7±18.2	23.5±8.7	48.3±17.5**	41.3±9.7*	0.42	0.70	1.21
Left	23.0±10.9	34.5±22.8	36.7±22.8	19.8±11.9	53.3±35.5	40.8±21.7	−0.28	0.63	0.18
rEn.MS/BS	−5.2±15.3	1.2±26.9	−13.0±18.0	3.7±8.7	−4.9±32.9	0.5±17.3	0.72	−0.20	0.76
**En.MAR (µm/day)**									
Right (loaded)	0.8±1.0	1.1±1.4	0.6±1.0	1.2±1.1	1.6±1.4	1.9±1.0	0.39	0.37	1.28
Left	0.6±0.6	0.6±0.8	1.4±1.1	0.4±0.8	1.7±0.8* a	1.3±1.0	−0.25	1.33	−0.07
rEn.MAR	0.2±1.1	0.5±1.7	−0.8±1.5	0.8±1.1	−0.1±1.0	0.5±0.8	0.54	−0.43	1.08
**En.BFR/BS (µm^3^/µm^2^/yr)**									
Right (loaded)	103±157	213±268	98±197	119±111	337±360	304±172	0.12	0.39	1.11
Left	48±53	97±186	239±234	45±86	392±300* a	247±213	−0.05	1.18	0.04
rEn.BFR/BS	55±138	117±277	−141±235	75±104	−56±295	57±196	0.16	−0.60	0.92
	***Humerus***								
**En.MS/BS (%)**									
Right	32.8±6.7	43.8±16.1	51.7±23.2	33.7±15.3	58.0±12.1**	39.8±10.6	0.08	1.00	−0.66
Left	36.2±10.1	49.7±13.3	42.8±9.4	29.8±12.4	52.2±10.0**	41.7±13.3	−0.57	0.22	−0.09
**En.MAR (µm/day)**									
Right	3.3±1.8	4.5±2.1	4.7±1.0	3.4±0.6	5.8±1.4***	4.9±0.7*	0.03	0.72	0.31
Left	4.9±2.6	4.9±2.4	4.5±1.4	3.3±0.8	5.7±0.8***	5.1±09**	−0.84	0.44	0.52
**En.BFR/BS (µm^3^/µm^2^/yr)**									
Right	412±273	794±523	853±296	397±142	1188±224***	702±152**	−0.07	0.98	−0.64
Left	688±503	950±586	698±308	353±143	1079±185***	776±286**	−0.91	0.31	0.26

**Note**: Data represent mean ± standard deviation.

* *p*<0.05;

** *p*<0.01;

*** *p*<0.001 versus the estrogen treated group.

a *p*<0.05 versus the associated Load group that did not receive brachial plexus anesthesia (BPA) before loading. Effect size comparing Load and BPA+Load groups for each respective treatment. En.MS/BS = endosteal mineralizing surface; En.MAR = endosteal mineral apposition rate; En.BFR/BS = endosteal bone formation rate. Relative (R-L) values were also calculated – rPs.MS/BS, rPs.MAR, and rPs.BFR/BS.

### Bone formation in response to loading was minimally altered after brachial plexus anesthesia

Within each estrogen treatment group, few significant changes were detected in load-induced bone formation after BPA. Comparisons between Load and BPA+Load groups for each treatment revealed a decreased bone formation in the Placebo+BPA group compared to the Placebo group in the ipsilateral humerus Ps.MS/BS (*p*<0.05), contralateral ulna En.MAR (*p*<0.05), and contralateral ulna En.BFS/BS (*p*<0.05). Effect sizes for rPs.MS/BS and rPs.BFR/BS were much larger in the estrogen-deficient (Placebo and G-1 groups), compared with the Estrogen group (**Table 2**).

### Estrogen-deficiency and gender influences mechanosensitivity to bone loading

When data were normalized with regard to applied strain, absolute mechanosensitivity and associated load-induced periosteal bone formation in young OVX female Sprague-Dawley rats after treatment with Estrogen, Placebo or G-1 was significantly lower than in young male rats of the same strain (*p*<0.001) (**Table 4**). OVX females treated with estrogen were also significantly less mechanosensitive when compared to OVX females treated with placebo and G-1 (*p*<0.01 and *p*<0.01, respectively) (**Table 4**). Male rats exhibited an adaptive response to the single-period loading that was significantly different from contralateral, whereas female rats did not. A similar result was found when data were also normalized for bone formation in the contralateral ulna. BPA blocking of Ps.L.B.Ar was also altered in the estrogen-deficient female rats (**Table 4**). The male rats used for this comparison (body weight 295–320 g, aged 70±2 days) were significantly younger than the female rats (*p*<0.001). There were no differences in rat age in the Estrogen, Placebo and G-1 groups.

**Table 4:** Mechanosensitivity to cyclic ulna loading in young female and male Sprague-Dawley rats.

Group	*Applied Peak Strain	*Frequency of applied load	Ulnar Ps.L.B.Ar (%/1,000 µε)	Ulnar rPs.L.B.Ar (%/1,000 µε)	Normalized rPs.L.B.Ar (%/1,000 µε)	BPA blocking of Ps.L.B.Ar (%/1,000 µε)	BPA blocking of Ps.BFR/BS (%/1,000 µε)
Male rats (n = 16)	−3,750 µe	4 Hz	5.65±0.84^a^	0.72±1.04#	0.046±0.056#	3.8	nd
OVX+Estrogen (n = 7)	−3,500 µe	2 Hz	1.68±0.66^b^	0.38±0.61	0.179±0.303	3.8	7.7
OVX+Placebo (n = 7)	−3,500 µe	2 Hz	2.90±0.86^c^	0.30±0.79	0.045±0.131	−3.2	−4.5
OVX+G-1 (n = 6)	−3,500 µe	2 Hz	2.91±0.35^c^	0.42±0.41	0.049±0.050	0.3	1.7

**Note**: Data represent mean ± standard deviation. OVX – ovariectomized females, G-1 – G-protein coupled estrogen receptor-specific agonist. Ps.L.B.Ar – periosteal labeled bone area *in the loaded ulna*, Ps.BFR/BS – periosteal bone formation rate, nd – not done, BPA – brachial plexus anesthesia. All rats were loaded for 1,500 cycles using a haversine waveform. Significant differences between groups (*p*<0.05) are indicated by different letters.

# Significantly different from a hypothesized mean of zero (*p*<0.05). Data from male rats previously published in Sample et al. 2008 [4].

* Because load-strain relationships were determined at 4 Hz and cyclic loading was applied at 2 Hz in females *in vivo*, viscoelastic effects in the ulna loading model [31] suggest applied peak strain in the female rats of the present study was likely equivalent to or greater than the male rats in the earlier work. rPs.L.B.Ar, = R – L. Normalized rPs.L.B.Ar = (R-L)/L [34]. BPA blocking of the loaded ulna = [1−(BPA+Load/Load)]×100 [4].

### Plasma estrogen levels were detectable in estrogen-treated rats but not estrogen deficient rats

Plasma estrogen levels were not detectable in the placebo and G-1 treated rats. In the estrogen treatment groups, the mean estrogen level was 7.14 pg/ml (range 0–25.1 pg/ml).

### Expression of ERα, ERβ, GPER, CGRPα, TRPV1, TRPV4 and TRPA1 in brachial intumescence dorsal root ganglia was minimally affected 10 days after mechanical loading in all groups

ERα gene expression was significantly decreased in the loaded limb brachial intumescence DRG of the Placebo group, when compared to the Estrogen group (*p*<0.05) at 10 days after loading. Relative (R-L) expression of ERα was increased after BPA in the Estrogen group. Expression of GPER in brachial intumescence DRG was significantly increased from internal control (trigeminal ganglion) in all samples (*p*<0.05) (**Table 5**). In the Estrogen group, DRG expression of ERβ was significantly decreased relative to internal control (p<0.05), whereas expression of ERα was higher than internal control. No significant differences were seen between groups for CGRPα, TRPV1, TRPV4 or TRPA1 gene expression at 10 days after loading. In all but one sample, expression of CGRPα was increased in DRG relative to trigeminal ganglion control (*p*<0.05). Expression of TRPA1 was significantly increased in trigeminal ganglion relative to DRG in all samples (*p*<0.05).

**Table 5:** Estrogen receptor expression in brachial intumescence (C_6_-T_2_) dorsal root ganglia gene.

	Load Groups			BPA+Load Groups		
	Estrogen	Placebo	G-1	Estrogen+BPA	Placebo+BPA	G-1+BPA
	(n = 8)	(n = 8)	(n = 7)	(n = 8)	(n = 7)	(n = 8)
**ERα**						
Right DRG	1.54±0.67	0.73±0.38*	1.30±0.79	1.57±0.47#	0.90±0.23	1.10±0.90
Left DRG	1.54±0.68	0.92±0.56	1.21±0.57	0.97±0.44	0.80±0.38	1.53±1.11
rERα (R-L)	0.00±0.39	−0.19±0.72	0.09±0.44	0.60±0.68^a^	0.11±0.41	−0.43±0.81
**ERβ**						
Right DRG	0.66±0.21#	0.94±0.76	0.90±0.36	0.92±1.06	0.85±0.31	1.06±0.62
Left DRG	0.67±0.33#	0.56±0.25#	1.13±0.89	0.67±0.47	0.71±0.21#	0.88±0.60
rERβ (R-L)	−0.01±0.32	0.39±0.79	−0.23±0.88	0.25±0.63	0.15±0.32	0.19±0.41
**GPER**						
Right DRG	2.12±1.16#	2.14±1.28#	2.79±1.42#	3.50±1.67#	3.35±1.48#	2.68±1.85#
Left DRG	2.73±1.30#	1.87±0.94#	3.70±2.31#	2.23±0.93#	2.48±0.59#	3.05±1.81#
rGPER (R-L)	−0.61±1.28	0.27±1.15	−0.91±2.44	1.27±1.86	0.87±1.73	−0.37±2.29

**Note**: Data represent mean ± standard deviation.

* *p*<0.05 versus the estrogen treated group.

# Significantly different from internal control (trigeminal ganglion) (*p*<0.05).

a Significantly different from a hypothesized mean of zero (*p*<0.05). DRG = dorsal root ganglia; ERα - estrogen receptor alpha; ERβ = estrogen receptor beta; GPER – G-protein couple estrogen receptor.

## Discussion

In this study we examined the effects of loading and BPA in ovariectomized female rats that were treated with either placebo, 17β-estradiol, or the GPER-specific agonist G-1. Our goal was to investigate whether load-induced bone formation was neuronally-regulated in female rats through an estrogen-dependent mechanism.

Estrogen has long been recognized as having important physiological effects on the skeleton. In particular, ERα signaling is thought to stimulate osteogenesis in response to bone loading, since ERα^−/−^ mice have a decreased response to bone loading compared to wildtype littermates [18], [35]. In contrast, signaling via ERβ leads to a reduced adaptive response to mechanical loading in females, since ERβ^−/−^ female mice have increased osteogenesis in response to bone loading, when compared to their wildtype littermates [19]. It is widely accepted that these estrogen signaling effects occur through direct ERα and ERβ signaling in bone cells. The non-genomic estrogen receptor GPER is also expressed in bone cells [36]. However, the physiologic role of GPER signaling in functional adaptation is unclear.

In the present study, rats in the Placebo and G-1 groups underwent OVX and were not given estrogen supplementation. We found animals in these groups formed a significantly greater amount of bone after mechanical loading than those rats in the Estrogen group that received 17β-estradiol. Increases in mineralizing surface in these treatment groups were particularly evident. The finding that rats in an estrogen-deficient state have increased bone formation after loading is not new, as estrogen deficiency from OVX enhances load-induced bone formation resulting from either direct bone loading or increased exercise [37], [38]. The suppressive effect of low-dose estrogen on load-induced periosteal bone formation is also found in young male rats [39]. The cellular mechanism that regulates the suppressive action of estrogen on load-induced periosteal formation has not been defined, although direct ERα- and ERβ-mediated effects in bone cells has been suggested [18], [19]. Mechanosensitivity to bone loading is also neuronally regulated in male rats [4]. Interestingly, it has recently been shown that ERα signaling in neuronal cells regulates bone mass, as conditional knockout mice in which ERα has been deleted in neuronal cells induces a high bone mass phenotype, suggesting a central inhibitory ERα signaling effect [40]. Therefore, the action of estrogen on mechanosensitivity and functional adaptation could be regulated via the nervous system.

To investigate whether the nervous system is involved in the physiological pathway through which estrogen regulates mechanosensitivity to bone loading, we performed BPA before ulna loading in additional groups of female rats. BPA had few significant effects on fluorochrome-labeled bone formation and no significant blocking effects were identified in the loaded ulna. This suggests that neuronal signaling effects on mechanosensitivity to bone loading are different in male and female rats. In male rats, central neuronal signaling acts to enhance adaptive responses to single-period bone loading [4], [5], whereas in the female rats in the present study, this effect was less evident, particularly in estrogen-deficient females. The effect of orchidectomy on BPA treatment in male rats has not been determined. An ulna loading protocol that would be expected to induce an adaptive response in male young rats did not consistently induce significant bone formation in the female rats in the present study.

To more directly compare gender differences in mechanosensitivity in young Sprague-Dawley rats, we calculated the bone formation normalized to applied peak strain in the female rats used in this study that were not given BPA, as well as male rats used in an earlier study in which a similar loading protocol was used [4]. Male rats had significantly greater absolute mechanosensitivity to loading than the Estrogen-, Placebo-, and G-1- treated female rats, although this effect was diminished when data were corrected for applied strain and contralateral bone formation.

Interestingly, the effect of BPA on bone formation was altered in estrogen-deficient females, particularly in the Placebo group. These observations fit with the hypothesis that central neuronal signaling appears altered if sex steroid deficiency is present. OVX in female rats leads to loss of sensory nerve fibers in bone and site-specific loss of bone mass at 14 days [16]. Collectively, these observations suggest that after OVX, central neuronal signaling effects on adaptive responses to bone loading changes from enhancement of load-induced bone formation to inhibition of load-induced bone formation.

The male rats used for this comparison were on average approximately 7 weeks younger than the female rats. This age difference may have contributed to gender differences in mechanosensitivity, as growth rate is also an important determinant of adaptive bone formation [34], [37], [38]. The rats of the present study were of a young adult age when growth rate reaches a plateau as mature body weight is reached [34]. In past work, normalization for growth rate has used the contralateral ulna [34]. However, since adaptive responses can involve contralateral bones [4], analysis of this effect using sham controls would be needed to fully separate adaptive responses and BPA treatment effects from underlying growth rate. Further work is needed to fully define gender and sex steroid effects on bone formation rate, mechanosensitivity to bone loading, and central neuronal signaling effects assessed by BPA. We found the group with the highest mean value was the OVX+estrogen group, when data are corrected for the underlying bone formation rate, suggesting that relative mechanosensitivity may actually be highest in intact females.

The role of the non-genomic estrogen receptor GPER on functional adaptation has not been previously determined. Female GPER^−/−^ mice have decreased bone growth [41]. GPER is also expressed in growth plate chondrocytes and is required for the normal estrogenic response in the growth plate in females [42], [43]. Our results suggest that estrogen signaling via the GPER receptor does not regulate the suppressive effect of estrogen on periosteal new bone formation.

Although we hypothesized that loading with or without BPA would influence gene expression in DRG of loaded and contralateral limbs, at 10 days after loading few changes in relative expression were evident. Estrogen is known to influence estrogen receptor signaling in DRG neurons [44]. Therefore, CNS plasticity to habitual loading of the skeleton [6] may include alterations to ERα expression. ERα, ERβ and GPER are all expressed in sensory neurons [45], [46], but only expression of GPER and CGRPα was consistently increased in DRG relative to trigeminal ganglion.

This study had several limitations. A priori, we had anticipated that the single period ulna loading protocol we used would consistently induce adaptive bone formation in female rats, as we found previously in male rats. However, we found that gender differences in absolute mechanosensitivity were much greater than anticipated and limited some of the conclusions that could be made from our results, particularly as non-loaded sham control groups [4] were not used in this study. Relatively small group sizes also limited statistical power. Another limitation is the retrospective nature of the comparison between males and females. Although viscoelastic effects associated with ulna loading [31] suggest that the magnitude of ulna loading in males and females was similar, use of a fully validated single-period loading protocol for male and female rats would be advantageous in future work. Examination of earlier time points after loading may have informed interpretation of gene expression analyses. Further work is needed to clarify whether estrogen signaling in DRG neurons influences the mechanosensitivity of the skeleton to bone loading and adaptive bone formation.

In conclusion, our data support previous studies showing that estrogen acts to decrease periosteal bone formation in female rats *in vivo*. This physiological effect is not GPER-mediated. The present study suggests that gender differences in absolute mechanosensitivity to bone loading exist in Sprague-Dawley rats, although sex steroid effects on bone formation rate and central neuronal signaling effects on load-induced bone formation [4] needs to be studied in more detail. Central neuronal signaling may form part of the mechanism that leads to bone loss after OVX in female rats.
